# RNA Sensors Enable Human Mast Cell Anti-Viral Chemokine Production and IFN-Mediated Protection in Response to Antibody-Enhanced Dengue Virus Infection

**DOI:** 10.1371/journal.pone.0034055

**Published:** 2012-03-30

**Authors:** Michael G. Brown, Sarah M. McAlpine, Yan Y. Huang, Ian D. Haidl, Ayham Al-Afif, Jean S. Marshall, Robert Anderson

**Affiliations:** 1 Department of Microbiology and Immunology, Dalhousie University, Halifax, Nova Scotia, Canada; 2 Department of Pathology, Dalhousie University, Halifax, Nova Scotia, Canada; 3 Department of Pediatrics, Dalhousie University, Halifax, Nova Scotia, Canada; 4 Canadian Center for Vaccinology, IWK Health Centre, Halifax, Nova Scotia, Canada; University of Tennessee Health Science Center, United States of America

## Abstract

Dengue hemorrhagic fever and/or dengue shock syndrome represent the most serious pathophysiological manifestations of human dengue virus infection. Despite intensive research, the mechanisms and important cellular players that contribute to dengue disease are unclear. Mast cells are tissue-resident innate immune cells that play a sentinel cell role in host protection against infectious agents via pathogen-recognition receptors by producing potent mediators that modulate inflammation, cell recruitment and normal vascular homeostasis. Most importantly, mast cells are susceptible to antibody-enhanced dengue virus infection and respond with selective cytokine and chemokine responses. In order to obtain a global view of dengue virus-induced gene regulation in mast cells, primary human cord blood-derived mast cells (CBMCs) and the KU812 and HMC-1 mast cell lines were infected with dengue virus in the presence of dengue-immune sera and their responses were evaluated at the mRNA and protein levels. Mast cells responded to antibody-enhanced dengue virus infection or polyinosiniċpolycytidylic acid treatment with the production of type I interferons and the rapid and potent production of chemokines including CCL4, CCL5 and CXCL10. Multiple interferon-stimulated genes were also upregulated as well as mRNA and protein for the RNA sensors PKR, RIG-I and MDA5. Dengue virus-induced chemokine production by KU812 cells was significantly modulated by siRNA knockdown of RIG-I and PKR, in a negative and positive manner, respectively. Pretreatment of fresh KU812 cells with supernatants from dengue virus-infected mast cells provided protection from subsequent infection with dengue virus in a type I interferon-dependent manner. These findings support a role for tissue-resident mast cells in the early detection of antibody-enhanced dengue virus infection via RNA sensors, the protection of neighbouring cells through interferon production and the potential recruitment of leukocytes via chemokine production.

## Introduction

Mast cells are well known for their classical role in inflammation and allergy but recent evidence has highlighted that their immune functions have much broader reaching implications [1], [2], [3], [4], [5], [6], [7], [8]. Studies suggest they also play an important sentinel cell role in host defence, with the capacity to specifically respond to various types of pathogens, including bacteria, fungi and viruses. Mast cells are abundant at mucosal sites and skin, placing them in an opportune location for interaction with invading pathogens.

Our studies involving mast cell responses to antibody-enhanced dengue virus infection have highlighted potent immunoregulatory activities of these cells, including secretion of tumor necrosis factor [9] and the chemokines CC chemokine ligand (CCL)3, CCL4 and CCL5 [10], [11]. These studies, in addition to other published reports [4], [6], [12], [13], reinforce the role of mast cells as innate immune effectors in response to virus infection. Furthermore, these studies provide insight into the diversity of signals generated in response to active virus infection or viral components, which can influence the mode/action of antiviral activity. Chemokines such as CCL3, CCL4 and CCL5 are important for the trafficking of leukocytes such as monocytes, T cells, and natural killer (NK) cells, all of which are suggested to play important roles in dengue infection. While the influence of CCL4 and CCL5 on the overall immune response to dengue virus infection is not well studied, clinically these chemokines are decreased in serum of dengue hemorrhagic fever patients, and therefore their levels may serve as good prognostic factors for disease outcome [14], [15].

Mast cells possess a complement of pattern recognition receptors that vary according to the host source and associated tissue or organ [12], [13], [16], [17], [18], [19], [20]. Human mast cells express the RNA sensor, Toll-like receptor (TLR)3 [13]. Recognition of viral dsRNA by mast cell TLR3 leads to signaling via TRIF to TBK1/IKKε to activate both interferon regulatory factor (IRF)3 and nuclear factor-κB (NF-κB) promoting the production of interferon stimulated genes, cytokines and chemokines. In the case of human mast cell line (HMC)-1, Laboratory of Allergic Diseases (LAD)-2 and primary CD34^+^ peripheral blood cell-derived mast cells, responses to extracellular polyinosiniċpolycytidylic acid (polyI:C) were shown to involve upregulation of type I interferons (IFNs) by RT-PCR [13]. Mast cells activated by polyI:C have also been reported to influence CD8^+^ T cell recruitment [12]. Furthermore, we have determined that polyI:C-exposed or reovirus-infected mast cells recruit NK cells in an CXCL8-dependent manner [21]. Additional studies have also indicated that polyI:C inhibits mast cell attachment to adhesion factors fibronectin and vitronectin [16].

The mechanisms by which dengue virus is detected by the innate immune system have begun to be investigated. Recently, St. John *et al.* demonstrated upregulation of retinoic acid inducible gene (RIG)-I and melanoma differentiation-associated protein (MDA)5 mRNA after dengue virus infection in a rodent mast cell line [22]. However, their model did not involve antibody-dependent enhancement, which is crucial for the interpretation of the role of mast cells in dengue hemorrhagic fever. RIG-I and MDA5 have also been shown to be important for IFN-β production in mouse embryonic fibroblasts [23] and possibly in A549 cells [24], [25]. Some evidence also exists to support the involvement of RIG-I or MDA5 in the induction of CCL5 and CXC chemokine ligand (CXCL)8 production [26], [27]. The dsRNA-dependent protein kinase (PKR) recognizes dsRNA and can mediate the inhibition of protein translation in response to type I IFNs and dengue virus dsRNA [28]. However, the function of PKR has not been investigated in mast cells. Together, all three sensors provide a mechanism by which the innate immune system induces the antiviral response when challenged by dengue virus.

In this study, we investigate human mast cell responses to antibody-enhanced dengue virus infection and exposure to the synthetic dsRNA analog (polyI:C). Since the link between the known virus sensors and subsequent mediator release has not been well characterized, these experiments will aid in identifying viral “triggers” of mast cell antiviral activity. This knowledge could potentially be exploited therapeutically to modulate host antiviral immunity.

## Materials and Methods

### Ethics Statement

Human umbilical cord blood was obtained without informed consent and the data were analyzed anonymously in accordance with the Izaac Walton Killam Health Center Research Ethics Board (certificate number 2808; Halifax, Canada). The requirement for consent was specifically waived by the Research Ethics Board.

### Cell culture

Human KU812 cells [29] were maintained in RPMI-1640 medium (Sigma, Oakville, Canada), supplemented with 10% fetal calf serum (FCS; Wisent, St-Bruno, Canada) and 10 mM HEPES. HMC-1 cells [30] were maintained in Iscove's modified Dulbecco's medium (Invitrogen, Burlington, Canada), supplemented with 10% FCS. All cell lines were passaged two to three times per week.

Primary human cord blood-derived mast cells (CBMC) were generated from umbilical cord blood mononuclear cells using a modification of the method of Saito *et al.* and characterized as previously described [31]. Umbilical cord blood was obtained from subjects undergoing elective caesarean sections with permission from the Izaac Walton Killam Health Center research ethics board (Halifax,Canada). Cells were cultured at an initial concentration of 0.6–1×10^6^ cells/mL and passaged weekly in RPMI-1640 medium supplemented with 20% FCS, 100 U/mL of penicillin, 100 µg/mL of streptomycin, 20% CCL-204 cell supernatant as a source of interleukin-6, 10^−7^ M prostaglandin E_2_ (Sigma), and 50 to 100 ng/ml of stem cell factor (Peprotech, Rocky Hill, NJ). For qPCR studies, CBMC cultures were sorted on a BD FACSAria (BD Biosciences, San Jose, CA) after at least 6 weeks of culture to enrich for high side scatter cells characteristic of mature mast cells. The sorted cells were returned to culture and the purity was assessed as described below. The purity of all cultures used was ≥95% as assessed by toluidine blue (pH 1.0) staining of cytocentrifuge preparations and examination of cells for the presence of multiple metachromatic granules and appropriate nuclear morphology.

### Reagents and conditions

The dsRNA analog polyI:C (Endogen, Thermo Scientific, Rockford, IL and Calbiochem, San Diego, CA) was used as a virus analogous stimulus. For transfection with polyI:C, 1 µg/ml was complexed with Lipofectamine 2000 as per the manufacturer's instructions (Invitrogen) and added to cells. Sheep anti-human IFN-α2b antibody (Endogen) and goat anti-human IFN-β antibody (R&D systems, Minneapolis, MN) were used at 1 or 10 µg/ml for blockade. Normal sheep or goat IgG were used as controls.

### Dengue virus propagation

Dengue virus, type 2 (strain 16681) [32] was propagated in African green monkey kidney Vero cell monolayers cultured in endotoxin-free RPMI-1640 medium (Sigma) supplemented with 1% FCS. In some cases, dengue virus was UV-inactivated by exposure to a germicidal lamp at a distance of 10 cm for 10 minutes.

### Sera

For antibody-dependent enhancement assays of dengue virus infection, a dengue-immune serum pool was prepared by using nine convalescent-phase sera from patients recovering from a dengue virus serotype 2 infection. The individual dengue-convalescent patient sera were obtained from a collection provided by Dr. Bruce Innis, Armed Forces Research Institute of Medical Science, Bangkok, Thailand [33].

### Infection and stimulation conditions

Prior to infection or stimulation, CBMCs were cultured overnight in normal culture medium lacking prostaglandin E_2_. Aliquots of infectious or UV-inactivated dengue virus were incubated alone or in the presence of a subneutralizing (1∶10,000) dilution of human dengue-immune serum for 60 min at 4°C. KU812, HMC-1 cells or CBMCs, were adsorbed with these inocula at a multiplicity of infection of 2–4 pfu/cell (as assayed on Vero cells) for 90 min at 4°C. Mock infection was performed using Vero cell-conditioned RPMI-1640 medium supplemented with 1% FCS. Unadsorbed virus and antibody was removed by washing once and cells were incubated in RPMI 1640 containing 1% FCS 37°C for 2, 6, 12, 24 or 48 hours as indicated. CBMCs were placed at 1×10^6^ cells/mL while HMC-1 and KU812 cells were incubated at 0.5×10^6^ cells/mL. In parallel conditions, CBMC, HMC-1 and KU812 cells were stimulated with 10 µg/mL polyI:C or with 1 µg/ml polyI:C complexed with Lipofectamine 2000. Cell-free supernatants were obtained by centrifugation at 300 g for 10 min and were frozen at −80°C.

### siRNA knockdown

KU812 cells (4×10^6^ cells/construct) were electroporated with 150 nM of siRNA specific for PKR, MDA5, RIG-I or a nonspecific, control sequence from Dharmacon (Lafayette, CO) using the Amaxa Nucleofactor Device according to manufacturer's instructions (Lonza, Rockland, ME). Two rounds of electroporation were performed 48 hours apart, followed by culture in RPMI containing 1% FBS. Six hours after the second electroporation, the cells were either mock treated or were infected with dengue in the presence of dengue-immune human serum. Knockdown was confirmed using the mock sample.

### Quantitative PCR (qPCR) and qPCR array analyses

Total RNA was isolated using the RNeasy mini kit (Qiagen, Frederick, MD) with DNase treatment (Qiagen) according to the manufacturer's protocol. One µg of total RNA was used for cDNA synthesis using a first strand cDNA kit (C-03 kit from SABiosciences/Qiagen) according to the manufacturer's instructions. Relative expression of a number of selected cytokine, chemokine and interferon-related genes, was determined using a custom human RT^2^ Profiler PCR Array (SABiosciences/Qiagen) with an RT^2^ SYBR Green/ROX qPCR Master Mix (SABiosciences/Qiagen) on a 7900HT Real-Time PCR system (Applied Biosystems, Carlsbad, CA). The fold-changes of gene expression, with GAPDH as internal control, were calculated using PCR array data analysis software (SABiosciences, Qiagen). For quantitative analysis of the mRNA expression of PKR, RIG-I, MDA5, CCL4, CCL8, and CXCL10, qPCR primer pairs for these genes were purchased from SABiosciences/Qiagen. cDNA was prepared as described above and qPCR was performed with the GoTaq qPCR Master Mix (Promega, Madison, WI) using the Stratagene MX3000P system and software (Stratagene/Agilent Technologies, Cedar Creek, TX). A PKR product was not successfully amplified from HMC-1 cDNA so PKR was therefore not included in the HMC-1 analysis. The Ct values for GAPDH and the genes listed above were used to calculate the normalized relative mRNA expression of each gene in virus-treated versus mock-treated samples according to the Pfaffl method [34].

### Chemokine Antibody Array

Chemokine content in supernatants from CBMCs stimulated with medium or dengue virus in the presence of dengue-immune sera as described above was analyzed using the human chemokine antibody array I from RayBiotech (Norcross, GA) according to the manufacturer's instructions. To prepare samples, 500 µL of supernatant from 2 separate donors were pooled before addition to the membrane. Membranes were exposed to Fujifilm Super RX Medical X-ray Film and signal intensity was quantified using Quantity One Software (Bio-Rad Laboratories, Mississauga, Canada). After blank subtraction from each spot, the positive controls were used to normalize individual spots across membranes. The fold increase in production in response to dengue+antibody/medium was determined and only those signals which were changed by ≥1.5-fold compared with medium were reported.

### Fluorescence-activated cell sorter (FACS) analysis

Cells were harvested at indicated times post-infection, fixed in 4% paraformaldehyde, permeabilized using 0.1% saponin and stained for intracellular expression of dengue E protein using monoclonal antibody 1B7 [35] or an immunoglobulin (Ig)G2a isotype control antibody. A goat anti-mouse Alexa 488 conjugate (Molecular Probes, San Diego, CA) was employed as a secondary antibody. Data were obtained using a FACScan flow cytometer (Becton Dickinson) and analyzed using FCS Express 3 (DeNovo Software, Los Angeles, CA).

### Enzyme-Linked Immunosorbent Assay (ELISA)

An ELISA kit was used according to the manufacturer's protocol to detect IFN-β (PBL Biomedical Laboratories, Piscataway, NJ). Sandwich ELISAs using commercial matched antibody pairs were obtained to further detect human IFN-α2b (Endogen), CCL4 (R&D Systems), CXCL8 (R&D Systems) and CXCL10 (BD Biosciences). CCL5 was detected by sandwich ELISA using a rabbit anti-human CCL5 antibody from Endogen with a goat anti-human secondary antibody from R&D Systems. ELISA plates were developed using the Invitrogen ELISA amplification system according to manufacturer's instructions, and were acquired on a Biotek Epoch microplate reader (Winooski, VT) and analysed using SoftMaxPro software (Molecular Devices, Sunnyvale, CA).

### Western blot

Cells were harvested at indicated times post-infection, washed in 1XPBS and immediately boiled for 5 min in SDS-containing loading buffer. Aliquots of 10 µg of total protein from samples were loaded and separated using 10% SDS-PAGE. Protein was then transferred to a PVDF membrane by semi-dry transfer. Blots were blocked in Tris-buffered saline containing 0.1% Tween 20+5% non-fat milk, washed and probed using polyclonal rabbit anti-human RIG-I (Cell Signaling Technologies, Danvers, CA), polyclonal goat anti-human MDA5 (Santa Cruz Biotechnology Inc., Santa Cruz, CA), monoclonal rabbit anti-human phospho-eIF2α (Ser51) (clone 1a19A11, Cell Signaling Technologies), rabbit polyclonal mouse anti-human eIF2α (Santa Cruz) and monoclonal mouse anti-mouse PKR antibodies (clone B10, Santa Cruz). After incubation with appropriate HRP-conjugated secondary antibodies, blots were developed with Super Signal West-Dura extended substrate kit (Pierce, Rockford, IL) and a Kodak Image Station 4000 mm Pro (Kodak, Rochester, NY).

### Statistical Analysis

All data are represented as the mean ± the standard error of the mean (SEM), unless otherwise stated. ELISA data were analyzed by ANOVA and paired one-tailed Student's t-tests. Data that were not normally distributed were analyzed by Friedman and Wilcoxon signed rank tests. Data sets that contained fewer than 6 experiments were analyzed by student's t-tests after log transformation. *P*<0.05 was considered significant.

## Results

### Mast cells upregulate multiple anti-viral signaling molecules, mediators and receptors in response to antibody-enhanced dengue virus infection

To more fully elucidate mast cell interactions with dengue virus, we used a qPCR array to broadly evaluate the genes upregulated by primary human CBMCs in response to antibody-enhanced dengue virus infection. The mRNA for several innate receptors was increased following dengue virus plus dengue-immune sera treatment, including PKR, RIG-I, MDA5, TLR3 and TLR7 (Table 1). Numerous mediator mRNAs were also upregulated such as type I IFNs and the chemokines CCL2, CCL3, CCL4, CCL5, CCL8, CXCL8 and CXCL10. Additionally, gene transcription of myxovirus resistance (MX)1, 2′-5′-oligoadenylate synthetase (OAS)1, OAS2, Myeloid differentiation primary response gene (MyD)88, NFKB1 and IRF7 was augmented. These responses were selective, as the quantity of mRNAs such as TLR8, IFN-αβR (IFNAR)1, IFNAR2, Jun, IRF3 and CCL11 did not change. Little change occurred when CBMCs were treated with UV-inactivated dengue virus in the presence of dengue-immune sera indicating that virus replication is necessary and that mere virus-antibody-receptor interaction is not sufficient to induce the observed responses.

**Table 1:** qPCR arrays reveal upregulation of anti-viral signaling molecules, receptors and mediators in dengue virus-infected CBMCs.

Gene	Fold-increase in mRNA expression/mock		Gene	Fold-increase in mRNA expression/mock	
	Dengue+Ab	UV-Dengue+Ab		Dengue+Ab	UV-Dengue+Ab
PKR	**11.3**	1.3	IRF7	**14.1**	1.4
OAS1	**108.0**	2.8	IRF9	**5.9**	1.4
OAS2	**243.2**	4.8	IFNAR1	1.0	1.0
MDA5	**25.3**	1.3	IFNAR2	0.8	1.2
RIG-I	**22.6**	1.0	IFNA4	**36.7**	0.7
LGP2	**17.4**	1.8	IFNA2	**303.4**	0.3
IPS-1	1.0	1.0	IFNB1	**366.1**	1.2
TLR3	**24.1**	1.2	MX1	**648.7**	9.4
TLR7	**3.8**	1.0	CCL2	**9.7**	2.2
TLR8	1.0	1.0	CCL3	**27.4**	2.4
TICAM1	**2.1**	1.1	CCL4	**78.5**	2.8
MYD88	**4.0**	1.1	CCL5	**76.4**	1.2
TOLLIP	1.1	1.0	CCL8	**9886.9**	11.4
NFKB1	**2.3**	1.1	CCL11	0.7	0.6
NFKB2	1.1	0.8	CCL17	1.2	0.9
JUN	0.9	1.3	CCL24	0.6	1.8
IRF1	**2.5**	1.0	CXCL8	**3.4**	1.9
IRF3	1.0	1.0	CXCL10	**93347.6**	43.0

Bold numbers indicate ≥2-fold higher levels of mRNA for the respective gene.

LGP2, laboratory of genetics and physiology 2; IPS-1, IFN-β promoter stimulator-1; TICAM1, Toll-like receptor adaptor molecule; TOLLIP, Toll interacting protein.

**Figure 1:**
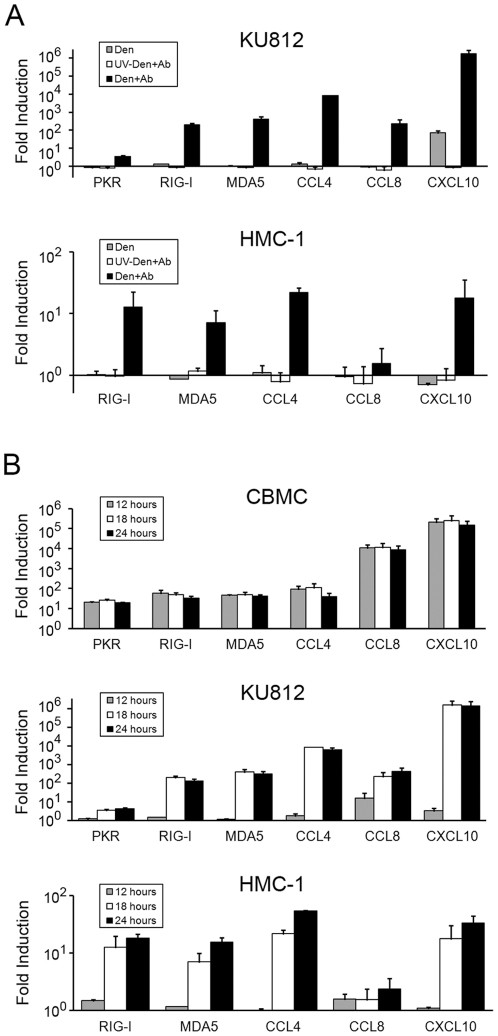
Quantitative PCR analyses of selected genes in response to antibody-enhanced dengue virus infection. A) KU812 and HMC-1 cells were exposed to infectious (Den+Ab) or UV-inactivated dengue virus (UV-Den+Ab) with a subneutralizing concentration of dengue-immune sera (1∶10,000). The cells were also exposed to dengue virus alone (Den) or were mock treated. At 18 hours post-infection RNA was prepared and qPCR was performed to analyze the mRNA levels of the indicated genes. After normalizing for the levels of GAPDH mRNA, the fold increases of the expression of RNA sensor and chemokine in virus-exposed cells relative to mock treated cells were calculated. Similar results were obtained at 24 hours post infection (not shown). Graphs show the mean ± SEM for KU812 (n = 3) and the mean ± SD for HMC-1 (n = 2). B) CBMCs, KU812, and HMC-1 cells were exposed to infectious dengue virus with a subneutralizing concentration of dengue-immune sera (1∶10,000) or were mock treated. At 12, 18, and 24 hours post-infection RNA was prepared and qPCR was performed to analyze the mRNA levels of the indicated genes. After normalizing for the levels of GAPDH mRNA, the fold increases in antibody-enhanced dengue-virus infected cells relative to mock treated cells were calculated. Graphs show the mean ± SEM for CBMC (n = 3 independent cultures) and KU812 (n = 3), in addition to the mean ± SD for HMC-1 (n = 2).

In order to study the changes in gene expression observed in the PCR array analysis in more detail, we performed qPCR analysis of the RNA sensors PKR, RIG-I, and MDA5 in addition to the chemokines CCL4, CCL8, and CXCL10. Multiple dengue virus infected CBMCs cultures in addition to the human mast cell lines KU812 and HMC-1 were analyzed. Similar to the results from the CBMC PCR array, gene induction in mast cells was dependent on viral replication (Figure 1A). In addition, the gene induction was largely dependent on the antibody-mediated enhancement of infection. Dengue virus alone did induce consistent upregulation of CXCL10 in KU812 cells, but this level was still greater than 20,000-fold less than the over one million-fold increase in CXCL10 mRNA after infection with dengue virus in the presence of antibody (Figure 1A). Interestingly, gene induction in HMC-1 cells was more modest relative to KU812, reaching a maximum increase of 22-fold for CCL4. A kinetic analysis of gene induction in three independent CBMC cultures showed a consistent, early, and prolonged upregulation of the RNA sensor and chemokine genes in response to antibody-enhanced dengue virus infection (Figure 1B). The fold transcriptional increase observed in infected CBMCs (ranging from approximately 20 for PKR to 250,000 for CXCL10) was similar to the increases seen in infected KU812 cells. In contrast to infected CBMCs, infected KU812 and HMC-1 cells showed little gene upregulation at 12 hours but revealed strong increases at 18 and 24 hours (Figure 1B).

**Figure 2:**
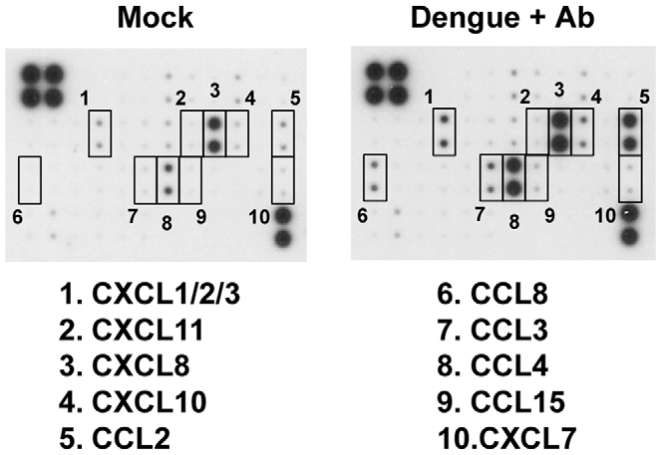
Antibody arrays reveal that CBMCs produce multiple chemokines in response to antibody-enhanced dengue virus infection. CBMCs were infected with dengue virus in the presence of a subneutralizing concentration of dengue-immune sera (1∶10,000) or were mock treated for 24 h, and the cell-free supernatants were analyzed for chemokine content using a chemokine antibody array. The most dramatically upregulated chemokines are indicated by numbered boxes. Results were obtained from two individual CBMC supernatants that were pooled prior to incorporation in the antibody array.

To confirm protein production of chemokines, supernatants from CBMCs infected with antibody-enhanced dengue virus or mock treatment were analyzed using a chemokine antibody array. Similar to the data presented in Table 1, the production of chemokines including CCL2, CCL3, CCL4, CCL8, CXCL8 and CXCL10 was selectively augmented after antibody-enhanced dengue infection compared with mock treatment (Figure 2 and Table S1). These data suggest that antibody-enhanced dengue virus-infected mast cells adopt an anti-viral phenotype that enables enhanced sensing and responsiveness to the invading virus.

### Mast cell production of chemokines is confirmed by ELISA

**Figure 3:**
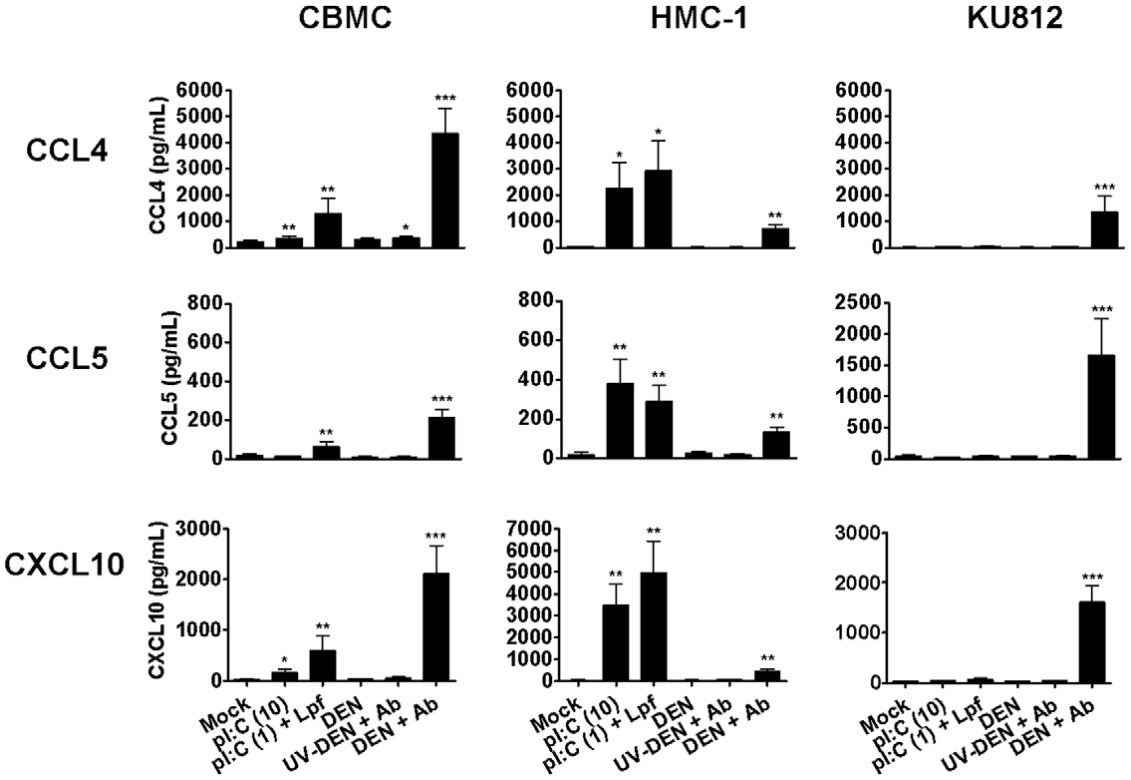
ELISA confirms CBMC antiviral chemokine release following antibody-enhanced dengue virus infection or polyI:C exposure. CBMCs, HMC-1 and KU812 cells were infected with dengue virus in the presence of a subneutralizing concentration (1∶10,000) of dengue-immune sera (DEN+Ab) or were exposed to 10 µg/mL polyI:C (pI:C (10)) or 1 µg/ml polyI:C complexed to Lipofectamine 2000 (pI:C (1)+Lpf). Controls included mock treatment, dengue virus only (DEN) and UV-inactivated dengue virus with dengue-immune sera (UV-DEN+Ab). After 24 hours, cell supernatants were harvested and measured for CCL4, CCL5 and CXCL10 by ELISA. Graphs show the mean ± SEM of 7–16 separate experiments. *P<0.05; **P<0.01; ***P<0.001.

To confirm our protein array findings, the production of chemokines by primary human CBMCs, HMC-1 and KU812 cells after antibody-enhanced dengue virus infection was evaluated by ELISA. PolyI:C was used as a surrogate viral RNA analog and was applied either exogenously or by transfection with Lipofectamine 2000. It was found that for CBMCs, polyI:C and antibody-enhanced dengue virus infection were able to induce significant levels of CCL4, CCL5 and CXCL10 (Figure 3). Transfected polyI:C was a more potent inducer of chemokine production than polyI:C applied exogenously. However, antibody-enhanced dengue virus infection induced the highest levels of chemokine compared with all other stimuli tested. CBMCs also produced 212 or 359 pg/mL of CXCL8 following transfection with polyI:C (*P* = 0.015) or antibody-enhanced dengue virus infection (*P* = 0.0073), respectively (n = 4–6).

Compared to CBMCs, HMC-1 cells responded to polyI:C with a similar CCL4, CCL5 and CXCL10 chemokine profile but at higher levels (Figure 3). In contrast, KU812 cells did not respond to either extracellular or lipid-transfected polyI:C (Figure 3). However, like HMC-1 cells, KU812 cells did produce CCL4, CCL5 and CXCL10 in response to antibody-enhanced dengue virus infection (Figure 3). Neither HMC-1 nor KU812 cells produced CXCL8 in response to any of the stimuli tested (n = 4–5).

### Time course analysis reveals that antibody-enhanced dengue virus infection results in the rapid CCL4 and CXCL10 production by CBMCs

**Figure 4:**
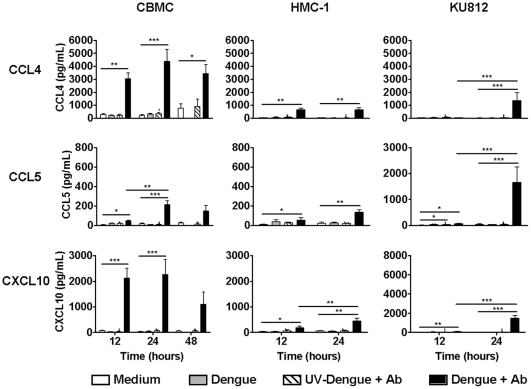
Time course analysis reveals rapid production of CCL4 and CXCL10 by antibody-enhanced dengue virus-infected CBMCs. CBMCs, HMC-1 and KU812 cells were infected with dengue virus with a subneutralizing concentration (1∶10,000) of dengue-immune sera (Dengue+Ab) for 12, 24, or 48 hours. Controls included mock treatment, dengue virus only (Dengue) and UV-inactivated dengue virus with dengue-immune sera (UV-Dengue+Ab). Cell supernatants were harvested and measured for CCL4, CCL5 and CXCL10 by ELISA. Graphs show the mean ± SEM of 3–16 separate experiments. *P<0.05; **P<0.01, ***P<0.001.

To examine the kinetics of chemokine production, CBMCs, HMC-1 and KU812 cells were infected for various times with dengue virus in the presence of dengue-immune sera and chemokine production was measured by ELISA. CBMC production of CCL4 and CXCL10 was near maximum at 12 hours and was still detected at 48 hours post-infection (Figure 4). CCL5 was produced more slowly and peaked at 24 hours. In contrast, but in agreement with the kinetics of chemokine mRNA induction (Figure 1), HMC-1 and KU812 cells produced CCL4, CCL5 and CXCL10 more gradually than CBMCs, with significant differences observed between 12 and 24 hours for all KU812 responses and CXCL10 production by HMC-1 cells. These data suggest a rapid chemokine response of human mast cells to antibody-enhanced dengue virus infection.

### Antibody-enhanced dengue virus infection and polyI:C exposure upregulates multiple RNA sensors in mast cells

**Figure 5:**
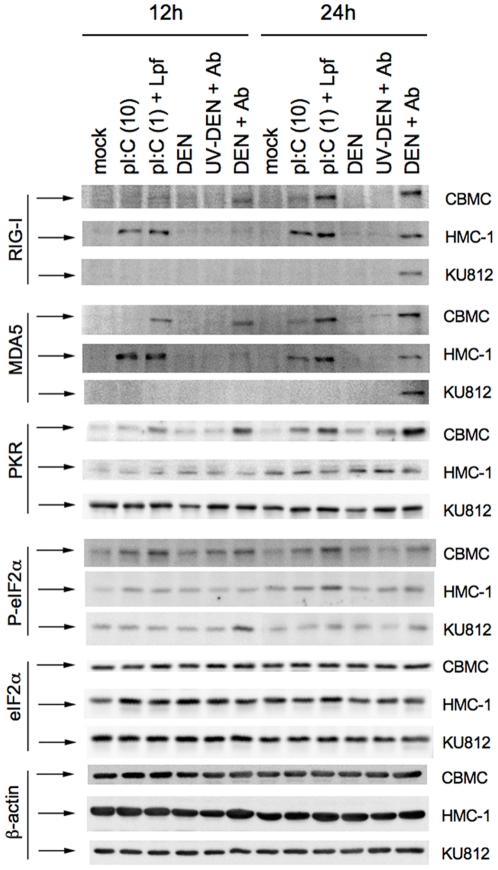
Mast cells upregulate RNA sensors following antibody-enhanced dengue virus infection and polyI:C exposure. CBMCs, HMC-1 and KU812 cells were exposed to infectious (Den+Ab) or UV-inactivated dengue virus (UV-Den+Ab) with a subneutralizing concentration of dengue-immune sera (1∶10,000), 10 µg/ml polyI:C (pI:C (10)) or 1 µg/ml polyI:C transfected with Lipofectamine 2000 (pI:C (1)+Lpf). As controls, CBMCs were exposed to dengue virus alone (Den) or were mock treated. At 12 and 24 hours post-infection cell lysates were prepared. A total of 10 µg of each lysate was resolved on a 10% SDS-PAGE and semi-dry transferred to PVDF and probed for RIG-I, MDA5, PKR, eIF2α and phospho-eIF2α, as well as β-actin as loading control. Data are representative of at least three separate experiments.

CBMCs, HMC-1 and KU812 cell lines were investigated for expression of the RNA sensors RIG-I and MDA5 following exposure to extracellular or transfected polyI:C or antibody-enhanced dengue virus infection (Figure 5). Upregulation of both RIG-I and MDA5 occurred in CBMCs in response to both transfected polyI:C and antibody-enhanced dengue virus infection at 12 hours post-infection, with some upregulation in response to extracellular polyI:C. Greater increases of RIG-I and MDA5 were observed by 24 hours post-infection. Upregulation of RIG-I and MDA5 protein levels in HMC-1 cells by extracellular and transfected polyI:C and antibody-enhanced dengue virus infection occurred, but with increased levels at the earlier 12 hour time-point, compared to CBMCs. Finally, while KU812 cells upregulated both RIG-I and MDA5 in response to antibody-enhanced dengue virus infection, neither extracellular nor transfected polyI:C had any effect on expression in these cells.

We investigated whether the well-known dsRNA receptor/kinase PKR was involved in the detection of polyI:C and dengue virus by CBMCs by employing western blot for total PKR protein and phosphorylation of the PKR substrate eukaryotic initiation factor (eIF)2α, indicative of PKR activation [36]. It was found that total PKR protein expression was increased at 12 hours in CBMCs following transfection with polyI:C or antibody-enhanced dengue virus infection (Figure 5). By 24 h, total PKR expression was further increased in these groups; in addition, an increase in response to extracellular polyI:C occurred. Phosphorylation of the host translation initiation factor eIF2α, which causes inhibition of host translation, occurred in response to extracellular and transfected polyI:C and antibody-enhanced dengue virus infection at both 12 and 24 h. Total eIF2α protein was unaffected. In contrast, HMC-1 and KU812 cells did not show the same upregulation of PKR and phosphorylated eIF2α.

### siRNA mediated knockdown of RNA sensors alters responses of KU812 cells to antibody-enhanced dengue virus infection

**Figure 6:**
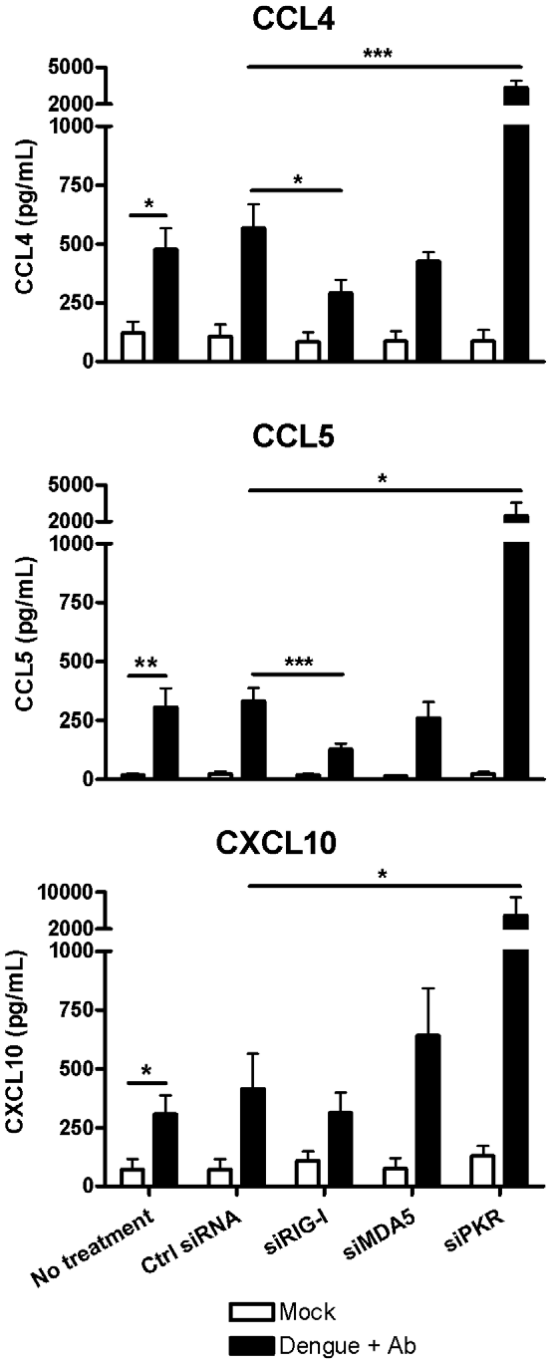
siRNA mediated knockdown of RNA sensors modulates KU812 responses to antibody-enhanced dengue virus infection. Following knockdown of the indicated genes by siRNA transfection, KU812 cells were mock treated or exposed to infectious dengue virus with subneutralizing concentration of dengue-immune sera (1∶10,000). At 24 hours post-infection supernatants were harvested and analyzed for CCL4, CCL5 and CXCL10 content by ELISA. Graphs show the mean ± SEM of 4 separate experiments. ns, not significant; *P<0.05; **P<0.01, ***P<0.001.

KU812 cells were used to investigate modulation of responses to antibody-enhanced dengue virus infection following siRNA mediated knockdown of the RNA sensors PKR, RIG-I and MDA5 (Figure 6). Knockdown of RIG-I led to a significant decrease in the amount of CCL4 and CCL5 chemokine production. In contrast, knockdown of PKR resulted in increased chemokine release, presumably due to the removal of eIF2α-mediated translational inhibition. Taken together, this suggests that RIG-I and PKR act as positive and negative regulators, respectively, of chemokine synthesis induced in response to antibody-enhanced dengue virus infection.

### Human mast cells infected with dengue virus secrete type I interferons that can potently suppress subsequent dengue virus infection

**Figure 7:**
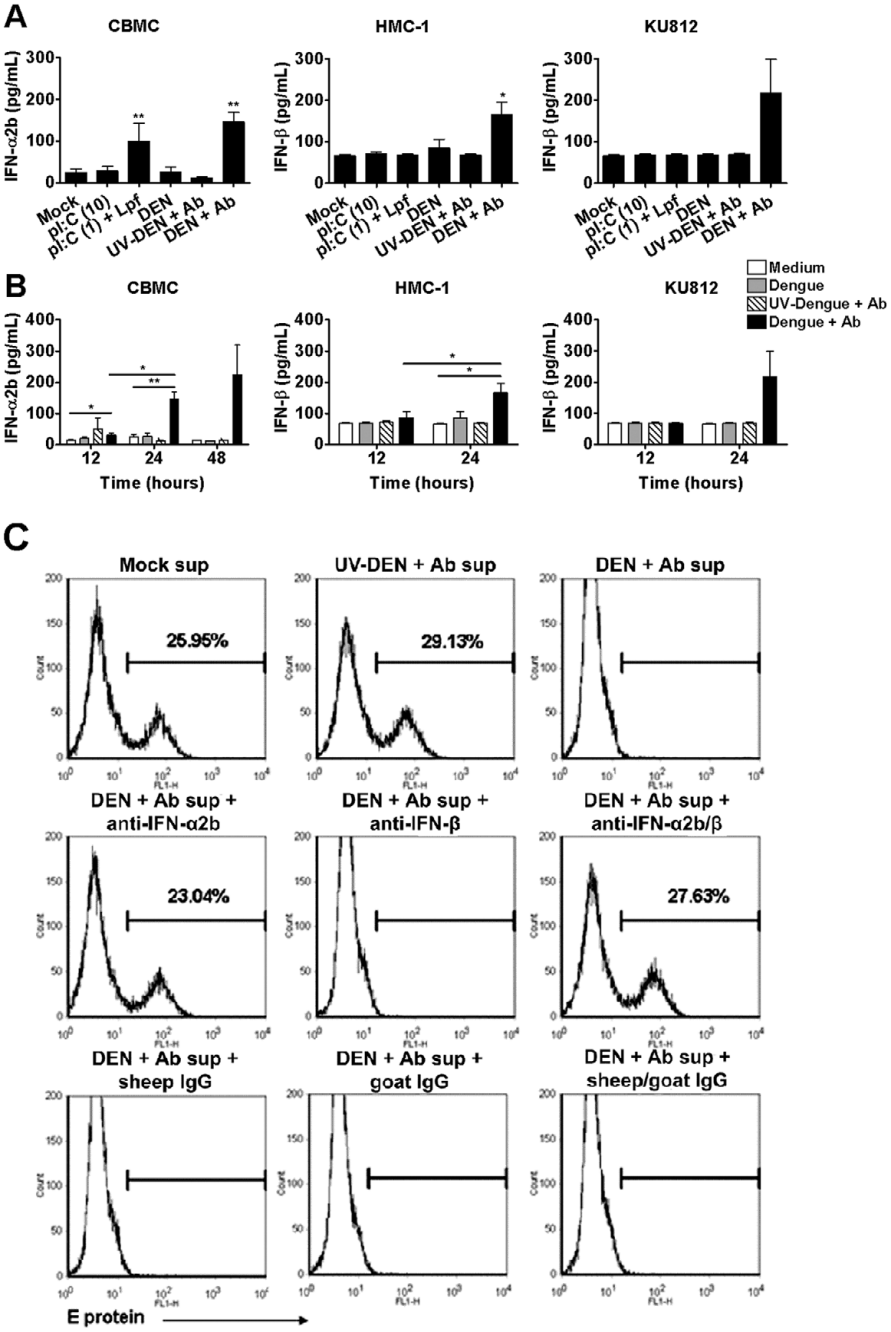
Type I interferons secreted by dengue virus-infected CBMCs suppress dengue virus infection of target cells. (A) CBMCs, HMC-1 and KU812 cells were infected with infectious (DEN+Ab) or UV-inactivated (UV-DEN+Ab) dengue virus in the presence of a subneutralizing concentration of dengue-immune sera (1∶10,000) or were exposed to 10 µg/mL polyI:C (pI:C (10)) or 1 µg/ml polyI:C complexed to Lipofectamine 2000 (pI:C (1)+Lpf). Controls included mock treatment and dengue virus only (DEN). After 24 hours, cell supernatants were harvested and assayed for IFN-α2b and IFN-β by ELISA. Graphs show the mean ± SEM of 4–9 separate experiments. (B) CBMCs, HMC-1 and KU812 cells were treated as in (A). After 12, 24 and 48 hours supernatants were analyzed for IFN-α2b and IFN-β content by ELISA. Graphs show the mean ± SEM of 3–9 separate experiments. (C) CBMCs were infected with dengue virus as described in (A). (C) 48 hour supernatants from CBMCs treated as in (A) were added directly or were blocked for 1 hour on ice with 10 µg/ml sheep anti-human IFN-α2b, goat anti-human IFN-β or appropriate control IgG, prior to addition to fresh KU812 target cells for 24 hours. Cells were then infected with dengue virus with a 1∶10,000 dilution of dengue- immune sera. At 24 hours post-infection KU812 cells were harvested, fixed and permeabilized for detection of intracellular dengue virus E protein by FACS. Data are representative of at least three separate experiments. *P<0.05; **P<0.01.

Since our initial mRNA array data clearly indicated an upregulation of mRNA for the anti-viral type I interferons in CBMC after antibody-enhanced dengue virus infection (Table 1), we examined mast cell type I IFN production and function in more detail. After 24 hours, CBMCs produced significant levels of IFN-α2b in response to transfected polyI:C and antibody-enhanced dengue virus infection (Figure 7A) but no IFN-β (data not shown). In contrast, HMC-1 and KU812 cells produced IFN-β only when infected with dengue virus (Figure 7A), and no detectable IFN-α2b. Time course analysis revealed that type I IFN production occurred gradually, with peak production observed at 24 to 48 hours (Figure 7B).

To examine the potential antiviral protective effects of the detected mediators, especially CXCL10 [37], [38] and type I IFNs, 48 h post-infection supernatants from mock- or variously treated CBMCs were added to fresh, uninfected target KU812 cells. Following incubation for 24 hours, the KU812 cells were then exposed to dengue virus in the presence of subneutralizing human dengue-immune serum. At 24 hours post-infection cells were analyzed for dengue envelope (E) protein by flow cytometry (Figure 7C). KU812 cells pretreated with supernatants from previously infected CBMCs were completely protected from antibody-enhanced dengue virus infection. In contrast, no protection was observed using supernatants from CBMCs which were mock-treated or treated with UV-inactivated dengue virus in the presence of dengue-immune sera. Neutralization of IFN-α2b using an IFN-α2b-specific antibody eliminated the supernatant-mediated protection of target KU812 cells from antibody-enhanced dengue virus infected CBMCs. This inhibitory effect was not observed with anti-IFN-β or control antibodies. Similar protective results were observed with HMC-1 and KU812 cell supernatants, and this was attenuated by addition of an IFN-β-specific antibody (data not shown). This implicates type I IFN as a major protective factor for viral infection produced by dengue virus-infected mast cells.

## Discussion

In this study we examined gene expression by primary human mast cells (CBMCs) and mast cell lines (HMC-1 and KU812) infected with antibody-enhanced dengue virus, and discovered that many key anti-viral receptors and mediators are upregulated during infection. We also showed that CBMCs, HMC-1 cells, and KU812 cells produce CCL4, CCL5, CXCL10 and type I IFNs when infected with dengue virus or stimulated with the dsRNA analogue polyI:C. Importantly, chemokine production by CBMCs occurred early in dengue virus infection. Mast cells increased expression of the RNA sensors RIG-I, MDA5 and PKR upon dengue virus infection or polyI:C exposure, and phosphorylation of the PKR substrate eIF2α indicated activation of the receptor. Moreover, siRNA-mediated knockdown of PKR, RIG-I and MDA5 demonstrated opposing roles for RIG-I and PKR in the regulation of chemokine production. Finally, mast cell supernatants were able to prevent infection of target KU812 cells, a process which was dependent on mast cell production of type I IFNs.

Interestingly, CBMCs produced only IFN-α at the protein level, whereas dendritic cells are known to produce both IFN-α and IFN-β when stimulated with polyI:C [39]. Selective mast cell production of IFN-α in the absence of IFN-β has also been confirmed in polyI:C-stimulated peripheral blood-derived mast cells [13]. Since the signaling pathways induced by polyI:C in dendritic cells are different from those triggered in endothelial cells and fibroblasts [40], it is possible that signaling in human mast cells and dendritic cells may also be distinct. The absence of MDA5 in uninfected mast cells may explain this difference, as resting dendritic cells do express this RNA sensor [41].

The expression of the cytosolic RNA receptors PKR, RIG-I, and MDA5 were found to all be increased in response to polyI:C and antibody-enhanced dengue virus infection. St. John *et al.* have also demonstrated increased expression of MDA5 and RIG-I in dengue virus infected mast cells in a rodent model that did not include antibody-dependent enhancement [22]. However, we did not observe increased expression with dengue virus alone (Figures 2 and 5), suggesting that the sensitivity of mast cells to dengue virus may vary by species. Our data showing the importance of RIG-I and PKR in regulating mast cell chemokine production advances our current understanding of RNA sensor function in mast cell responses to viral infection (Figure 6).

Our findings provide the first demonstration that mast cells can produce sufficient amounts of type I IFNs to protect neighbouring cells from infection. The induction of an anti-viral state by type I IFNs is a well-known initial response to viral infection [42]. Type I IFNs have been demonstrated to increase PKR, RIG-I, and MDA5 expression along with a number of other important interferon stimulated genes [43], [44], [45]. The upregulation of RNA sensors such as RIG-I and MDA5 appears to be key for the suppression of dengue virus replication [25], [46], [47]. The possibility that tissue-resident mast cells can initiate this vital response places them in a position to protect neighbouring cells at the tissue site rapidly after inoculation. The establishment of an antiviral state also supports our findings that PKR is activated in mast cells upon dengue virus infection, as protein translation inhibition during virus infection is dependent on the PKR substrate, eIF2α [28].

Circulating levels of CCL4 and CCL5 in dengue patients are positively correlated with good prognosis [14], [15]. This may be explained by their chemoattractant function for T cells and NK cells, both of which are important for viral clearance. Additionally, in mouse models of dengue infection, CXCL10 is crucial for CD8^+^ T cell and NK cell infiltration to sites of viral replication. Mice lacking the CXCL10 receptor CXCR3 have increased mortality rates compared to wild type [37], [38]. High concentrations of CXCL10 have also been shown to inhibit dengue virus entry by competing for heparin sulfate binding [37]. Conversely, CXCL8 and CXCL10 appear to be an indicator of increased disease severity and patient death [48], [49], of which the former may be due to CXCL8-induced vascular permeability [50]. These studies suggest that mast cell production of CCL4, CCL5 and CXCL10 may be beneficial in the immunity to dengue virus, however more studies will be required to understand the overall effects of mast cell responses in dengue virus infection.

The ability to respond to polyI:C, either extracellular or cytosolic, varied among the mast cells examined. While HMC-1 cells responded to both extracellular and transfected polyI:C, CBMCs responded mainly to transfected polyI:C, and KU812 cells were unresponsive to both. However, antibody-enhanced dengue virus infection was able to stimulate mediator release and RNA sensor upregulation in all three types of mast cells. These results most likely reflect a varied RNA sensor repertoire between different mast cells. The endosomal RNA sensors TLR3, TLR7 or TLR8 may participate in the detection of extracellular polyI:C and dengue virus. TLR3 may be important for the detection of dengue virus by certain cell types, including monocytic cell types [51]. Human peripheral blood-derived mast cells and the HMC-1 mast cell line have previously been shown to respond to extracellular polyI:C resulting in type I IFN production via TLR3 [13]. However, TLR3 protein expression and function in CBMCs and KU812 cells, remains to be determined.

The inability of KU812 cells to respond to transfected polyI:C is a curious finding, since KU812 cells are able to express PKR, RIG-I and MDA5, with PKR occurring at high constitutive levels (Figure 5). One consideration may be the preparation of polyI:C used for these studies. While PKR is reported to bind dsRNA greater than 30 nucleotides, the requirements for recognition of polyI:C by MDA5 and RIG-I remain controversial. RIG-I binds to 5′-triphosphorylated RNA and dsRNA shorter than 1 Kb, while MDA5 binds dsRNA longer than 2 kb [52]. Our source of polyI:C is reported by the manufacturer (Endogen) to range between 2–3 kb. Future studies examining a range of verified polyI:C lengths, *in vitro* transcribed RNA and those with 5′-triphosphates may provide insight into the responsiveness of mast cells to various virus analogous stimuli.

The early detection of invading pathogens is critical in preventing more serious manifestations of disease [53]. Time course analysis revealed that primary human CBMCs accumulate maximal levels of CCL4 and CXCL10 mRNA and produce CCL4 and CXCL10 in nanogram amounts as early as 12 hours post-infection (Figures 1, 3, and 4). Few studies have demonstrated chemokine production this early in infection. While monocyte-derived dendritic cells can produce large amounts of CCL4 [54] and CXCL10 [55] in response to dengue virus, they are not resident in the dermis as mast cells are, and would not be activated immediately upon dengue virus infection. Therefore, mast cells are likely key responders in the detection and mobilization of the immune system during the early stages of dengue virus infection.

Our data support a role for mast cells in the protection of neighbouring cells through type I IFN production. Furthermore, human mast cell-derived chemokines may recruit key effector cells such as NK cells and T cells to the site of infection. In support of this, a recent *in vivo* study using a mouse model of infection demonstrated that mast cells are important for NK cell and NK1.1^+^ T cell recruitment to dengue virus infection sites [22]. It should be noted however that mast cell production of mediators such as CCL4 may also attract macrophages, which are primary targets of dengue virus infection. Further studies focusing on the impact of mast cell mediators on surrounding cell populations during dengue virus infection will provide insight into the true impact of their responses.

This report is the first study demonstrating an important role for RNA sensors, particularly RIG-I and PKR, in human mast cell cytokine/chemokine responses to dengue virus. Mast cells are tissue-resident granulocytes with the ability to selectively produce profiles of mediators, and are likely first responders in infection. These findings broaden our knowledge of mast cell responses to dengue virus and virus infection in general and provide strong support for the role of mast cells as innate sensors of pathogen invasion.

## Supporting Information

Table S1 **Chemokine production by dengue virus-infected CBMCs analyzed by antibody array.**
(DOC)
