# Supplementary material for: RNA Sensors Enable Human Mast Cell Anti-Viral Chemokine Production and IFN-Mediated Protection in Response to Antibody-Enhanced Dengue Virus Infection
Source: PLoS One. 2012 Mar 30;7(3):e34055. doi: 10.1371/journal.pone.0034055 (PMC3316603; doi:10.1371/journal.pone.0034055)
Supplement: Table S1 — Chemokine production by dengue virus-infected CBMCs analyzed by antibody array. (DOC) [file pone.0034055.s001.doc]

Table S1. Chemokine production by dengue virus-infected CBMCs analyzed by antibody array.

| **Chemokine** | **Fold Change** |
| --- | --- |
| CCL2 | 12.0 |
| CCL3 | 9.5 |
| CCL4 | 5.0 |
| CCL8 | NQ |
| CCL15 | 3.1 |
| CXCL1/2/3 | 3.1 |
| CXCL7 | 1.8 |
| CXCL8 | 1.8 |
| CXCL10 | 3.8 |
| CXCL11 | 2.1 |

1. Results were calculated by determining the fold-increase or –decrease in response to dengue virus with dengue-immune sera compared with mock. The arrays were performed using pooled samples from two separate CBMC activations.

2. NQ indicates not quantified due to lower than background spot density on the mock array.
